# Bio‐inspired ionic skins for smart medicine

**DOI:** 10.1002/SMMD.20220026

**Published:** 2023-02-12

**Authors:** Zhouyue Lei, Wentao Xu, Guogao Zhang

**Affiliations:** ^1^ John A. Paulson School of Engineering and Applied Sciences Harvard University Cambridge Massachusetts USA

**Keywords:** bio‐inspired materials, healthcare, hydrogels, ionic skins, smart medicine

## Abstract

Ionic skins are developed to mimic the mechanical properties and functions of natural skins. They have demonstrated substantial advantages to serve as the crucial interface to bridge the gap between humans and machines. The first‐generation ionic skin is a stretchable capacitor comprising hydrogels as the ionic conductors and elastomers as the dielectrics, and realizes pressure and strain sensing through the measurement of the capacitance. Subsequent advances have been made to improve the mechanical properties of ionic skins and import diverse functions. For example, ultrahigh stretchability, strong interfacial adhesion, self‐healing, moisturizing ability, and various sensing capabilities have been achieved separately or simultaneously. Most ionic skins are attached to natural skins to monitor bio‐electrical signals continuously. Ionic skins have also been found with significant potential to serve as a smart drug‐containing reservoir, which can release drugs spatially, temporally, and in a controllable way. Herein, this review focuses on the design and fabrication of ionic skins, and their applications related to smart medicine. Moreover, challenges and opportunities are also discussed. It is hoped that the development of bio‐inspired ionic skins will provide a paradigm shift for self‐diagnosis and healthcare.

## Graphical abstract

Bio‐inspired ionic skins are inspired by natural skins and developed to bridge the gap between machines and human bodies. This review discusses the molecular design and healthcare applications of ionic skins. We believe the development of bio‐inspired ionic skins will reshape the future of smart medicine.

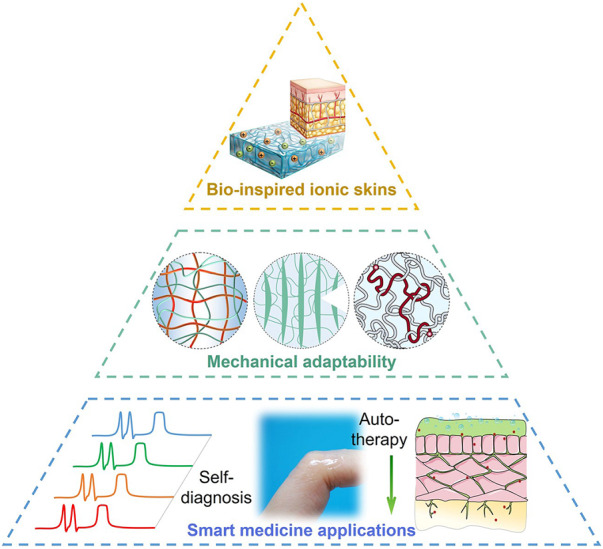

1

**Key points**

- Fundamental concepts of ionic skins are discussed.
- The medicine applications of ionic skins are discussed.
- Future directions of next‐generation ionic skins for personalized healthcare are presented.

## 2 INTRODUCTION

Natural skins are soft, stretchable, and able to sense diverse external stimuli, such as touch, vibration, heat, and cold.^1^, ^2^, ^3^ Benefiting from the recent advances in flexible electronics, artificial skins realizing similar mechanical behaviors and sensory capabilities have been emerging.^1^, ^4^, ^5^, ^6^ Artificial skins are generally classified as ionic skin and electronic skin depending on the species of the charge carriers, with the former using ions as the charge carriers and the latter electrons.^2^, ^6^, ^7^, ^8^, ^9^, ^10^, ^11^ Ionic skins are made of ionic conductors, such as hydrogels, whereas electronic skins are usually made of metals, conducting polymers, and carbon materials. Ionic skins, therefore, show some advantages compared with electronic skins, including softness, intrinsic stretchability, high transparency, and elimination of possible electrochemical reactions.^6^, ^10^, ^12^ They are sensory sheets that report signals using ions as the charge carriers.^6^ The sensing capability is usually realized by the migration of ions upon various stimuli, including pressure, deformation, and temperature. These ionic signals can be converted into electrical signals through ion–electron interfaces mainly through double layer charging instead of a Faradaic reaction.^13^ Afterward, the output electrical signals transport through external circuits to a storage or a processor for signal recording and analysis.

Some materials have been found with outstanding ionic conductivity, such as liquid electrolytes, salt‐containing hydrogels, and ionomers, therefore all of them are valid choices for fabricating ionic skins.^13^, ^14^, ^15^ For most of the existing ionic skins, hydrogels are chosen as the ionic conductors because of their biocompatibility, structural diversity, and relatively high conductivity of small ions and molecules.^6^, ^8^, ^9^, ^10^, ^16^ For example, regular hydrogels have water content similar to the biological tissue, providing an excellent extracellular physiological environment.^17^, ^18^, ^19^, ^20^ Hydrogels of hierarchical structures have been prepared to mimic the structures of natural skin, achieving good mechanical properties.^21^, ^22^, ^23^ Hydrogels swollen with drugs have the potential to realize on‐demand delivery of drugs.^20^, ^24^, ^25^, ^26^ We will further discuss the molecular design of hydrogels in this review.

Despite the development of ionic skins, challenges remain in the long‐term stability of the ionic signals, integration of data processing components, and portable power supplies. Herein, we review (1) the molecular design of hydrogels that improves the mechanical adaptability of the ionic skins, (2) the specific advantages of ionic skins in the application of smart medicine, and (3) the remaining challenges and possible solutions. It is hoped that ionic skins can bring new opportunities for smart medicine.

## 3 DESIGN OF BIO‐INSPIRED IONIC SKINS

**FIGURE 1:**
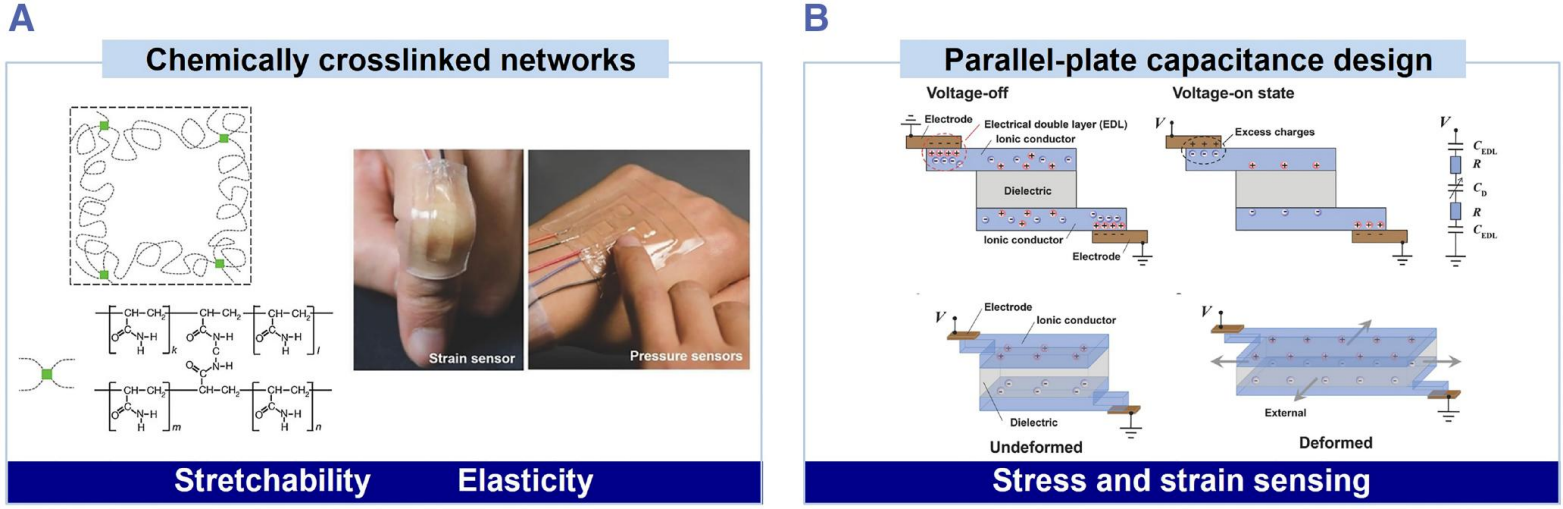
The design of first‐generation ionic skins. Reproduced with permission.^6^ Copyright 2014, John Wiley and Sons. (A) Chemically cross‐linked networks and a stretchable transparent device. (B) Parallel‐plate capacitance configuration and its sensing mechanism.

The first‐generation ionic skin is a parallel‐plate capacitor, formed by two layers of polyacrylamide hydrogels as the conductors and a layer of a stretchable insulating elastomer in between as the dielectric.^6^ Both the chemically cross‐linked hydrogel and the elastomer are stretchable and biocompatible, and so is the ionic skin (Figure 1A). Both polymers are safe and biocompatible in vitro.^6^, ^7^ The polyacrylamide hydrogel contains hydrated salts, allowing ionic conductivity.^6^ When a small voltage is applied between the two hydrogels, a parallel‐plate capacitor and two electrical‐double‐layer capacitors are formed and connected in series (Figure 1B). Because the electrical‐double‐layer has higher capacitance than the parallel‐plate capacitor, the overall capacitance of the ionic skin is roughly equal to the capacitance of the parallel‐plate capacitor. The ionic skin could detect deformation ranging from 1% to 500%, indicated by the change in capacitance.^6^ The ionic skin is both stretchable and transparent, allowing special applications that are hard to realize with other materials.^27^, ^28^, ^29^ In addition to the parallel‐plate capacitor, other device configurations, such as resistors and electrical‐double‐layer capacitors, are also used to fabricate ionic skins with enhanced tactile and pressure sensitivity.^8^, ^30^, ^31^, ^32^

## 4 MOLECULAR DESIGN OF HYDROGELS

Regular hydrogels are prepared from precursors of water, monomers, initiators, and cross‐linkers. The monomers are initiated by initiators and interconnect to form polymer chains. The polymer chains are chemically cross‐linked by cross‐linkers to form a network, which imbibes a large amount of water.^7^

**FIGURE 2:**
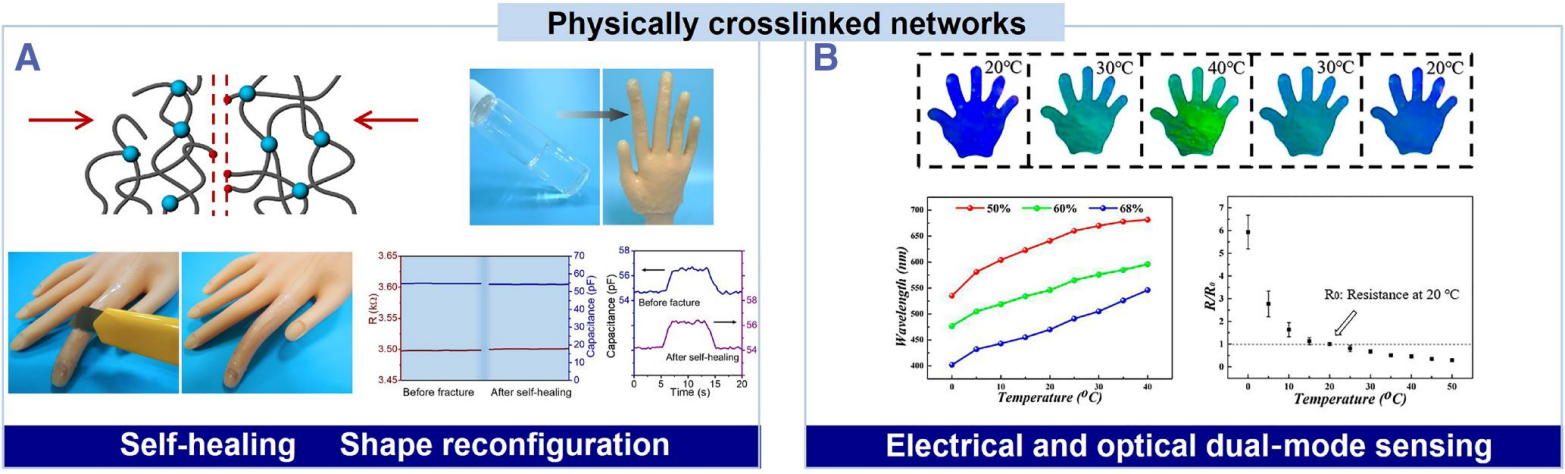
Physically cross‐linked network design of self‐healing and chromatic ionic skins. (A) Physically cross‐linked networks and a self‐healing device. Reproduced under terms of the Creative Commons Attribution 4.0 International License.^33^ Copyright 2018, The Authors, published by Springer Nature. (B) Electrical and optical dual‐mode sensing by filler alignment. Reproduced with permission.^34^ Copyright 2020, National Academy of Sciences.

A physically cross‐linked hydrogel is cross‐linked by non‐covalent interactions, such as hydrogen bonds, ionic associations, hydrophobic interactions, and coordination interactions.^30^ Physically cross‐linked hydrogels can be used to fabricate ionic skins of self‐healing, tunable mechanical properties, and special optical functions (Figure 2). These non‐covalent interactions can undergo bond breakage and reform, enabling self‐healing (Figure 2A).^33^, ^35^ In addition, the strength of the non‐covalent interactions differs and is usually much lower than that of covalent bonds with some exceptions.^33^, ^36^ The diversity in the strength of the non‐covalent interactions is beneficial for fabricating ionic skins of adjustable viscoelasticity and tunable modulus, which are crucial for the robustness of the interface. The physically cross‐linked hydrogels also enable electrical and optical dual‐mode sensing using polymer chain arrangement and potential filler alignment (Figure 2B).^34^, ^37^, ^38^, ^39^, ^40^, ^41^, ^42^, ^43^, ^44^

**FIGURE 3:**
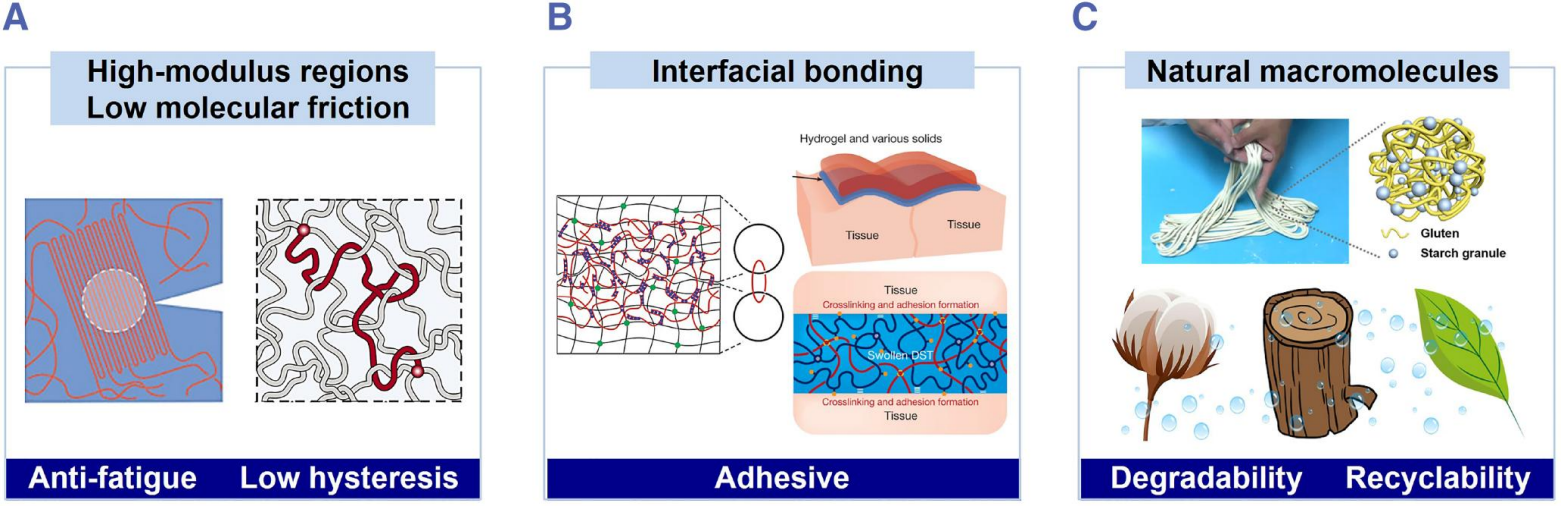
Molecular design of hydrogels to import new functions and improve the mechanical adaptability of ionic skins. (A) Tough and low‐hysteresis hydrogels. Reproduced under terms of Creative Commons Attribution NonCommercial License 4.0 (CC BY‐NC).^21^ Copyright 2019, The Authors, published by the American Association for the Advancement of Science. Reproduced with permission.^22^ Copyright 2021, The American Association for the Advancement of Science. (B) Interfacial bonding. Reproduced with permission.^45^ Copyright 2018, John Wiley and Sons. Reproduced with permission.^46^ Copyright 2019, Springer Nature. (C) Degradable and recyclable hydrogels. Reproduced with permission.^47^ Copyright 2020, John Wiley and Sons.

Tough hydrogels are used to fabricate ionic skin with improved fracture behaviors (Figure 3A). Natural skins are stiff, strong, and tough, whereas regular hydrogels are not. To meet the challenges, several efforts have been made to improve the mechanical properties. Double network hydrogels achieve improved toughness through energy dissipation of the sacrificial bonds.^17^, ^48^ However, this energy dissipation will cause high mechanical hysteresis, leading to increasing signal drift and declining mechanical properties during cyclic loading. Tough hydrogels of low hysteresis are desired to meet the requirements of practical applications. Hydrogels of dense entanglements achieve high toughness and low hysteresis simultaneously, as well as high stiffness and high fatigue threshold.^21^, ^22^, ^49^, ^50^, ^51^, ^52^ Compositing represents another general method to enhance the mechanical properties of hydrogels. For example, hydrogels of hierarchical structures show exceptional mechanical properties.^21^, ^22^, ^23^, ^53^, ^54^, ^55^ Inorganic/organic fillers embedded in hydrogels, such as graphene oxide, MXene, and microgels, prohibit crack propagation by stress deconcentration at the filler–matrix interface, and increase the stretchability and strength of the hydrogels.^56^, ^57^, ^58^, ^59^

The strong interfacial adhesion between hydrogels and natural skin is also crucial for the performance of the ionic skin (Figure 3B).^45^, ^60^, ^61^, ^62^, ^63^ The epidermis, the outermost layer of skin, is rich in amino acids, which can serve as hydrogen‐bonding donors and acceptors. By taking advantage of this, hydrogels of moderate adhesion with natural skins (<50 J m^−2^) are prepared.^64^ To increase the adhesion, other mechanisms are considered. For example, catechol groups are introduced into hydrogels to promote adhesion through hydrogen bonding and ionic associations.^31^, ^65^ By coupling the amino acids in biological tissues covalently with some polyelectrolyte hydrogels, the adhesion toughness >710 J m^−2^ can be achieved on wet porcine skin.^46^, ^66^ Besides, topological entanglement improves the adhesion between two non‐adhesive interfaces through the diffusion and in situ cross‐linking of a third polymer network.^45^, ^67^, ^68^, ^69^

Degradable and recyclable hydrogels can contribute to conserving resources and protecting the environment. With the increasing demand for wearable devices, the resulting electronic and plastic wastes will cause environmental pollution. To alleviate environmental issues, transient and biodegradable electronics are developed.^70^, ^71^ As for ionic skins, many hydrogels can be prepared from many natural materials, such as cellulose and proteins, which can be readily recycled and reused (Figure 3C). For example, natural dough is used to fabricate ionic skins to monitor body movements and can be recycled above 10 times without sacrificing the mechanical and sensing properties.^47^ With the development of functions and optimized mechanical properties, recyclable materials will attract much more interest than conventional materials.

## 5 SMART MEDICINE APPLICATIONS

**FIGURE 4:**
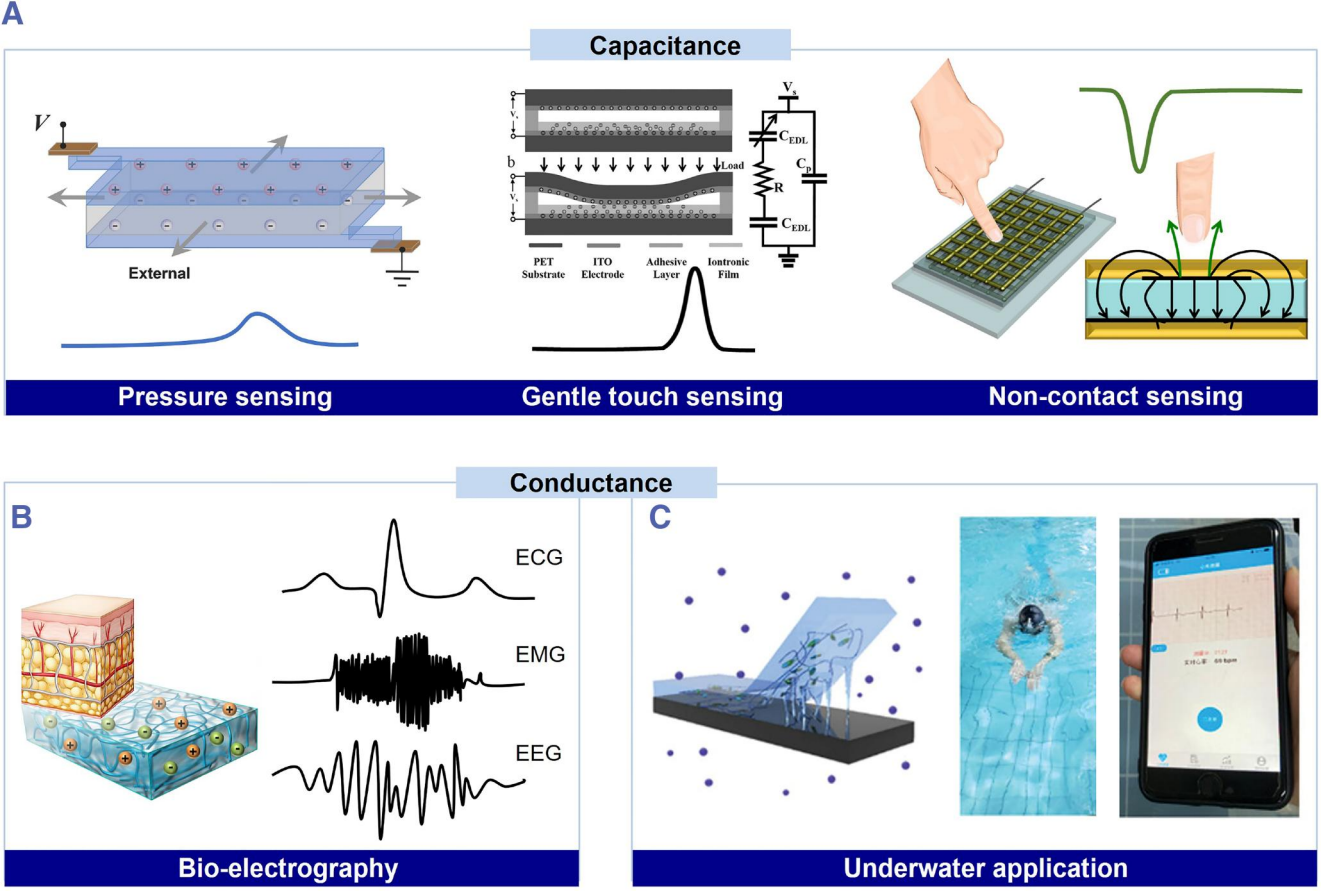
Self‐diagnostic applications of bio‐inspired ionic skins via capacitance and conductance. (A) Monitoring body movements through various capacitance signals. Reproduced with permission.^6^ Copyright 2014, John Wiley and Sons. Reproduced with permission.^8^ Copyright 2015, John Wiley and Sons. Reproduced with permission.^72^ Copyright 2019, Royal Society of Chemistry. (B) Acquirement of physiological signals like electrocardiography (ECG), electromyography (EMG), and electroencephalography (EEG) via ionic conducting. (C) Underwater application of ionic skin enabling remote monitoring. Reproduced with permission.^56^ Copyright 2021, John Wiley and Sons. Reproduced with permission.^73^ Copyright 2021, John Wiley and Sons.

Ionic skins enable self‐diagnosis in vitro through the measurement of capacitance, conductance, voltage, resistance, etc. To detect body movements, a variety of capacitance signals that are barely affected by temperature and humidity are preferred (Figure 4A). For the ionic skins constructed in parallel‐plate capacitance configuration, stress induced by body movements causes the deformation of the capacitor.^6^ The ionic skins thus monitor body movements through the correlation between the strain and capacitance. In addition to parallel‐plate capacitors, electric‐double‐layer capacitors show much higher sensitivity to small pressure, which is more favorable for gentle touch detection.^8^, ^74^, ^75^, ^76^ Besides, fringing capacitance enables noncontact sensing of a grounded conducting medium, such as a finger. When a finger approaches the ionic skin, the fringing electric field is partially intercepted and shunted to the ground by the finger, leading to a decrease in capacitance.^72^, ^77^

High adhesion and ionic conductivity (about 10 S m^−1^) are beneficial to eliminate interfacial impedance for long‐term monitoring of bio‐electrography, such as electrocardiography (ECG), electromyography (EMG), and electroencephalography (EEG) (Figure 4B).^78^ These signals are usually collected through metal electrodes attached to the human body. These rigid metal electrodes may cause allergies, and it is hard for them to achieve conformal and seamless contact with the human body.^2^ Ionic skins based on hydrogel conductors readily solve these issues through their excellent biocompatibility and softness.^20^ For example, mussel‐inspired adhesive ionic skins are developed to acquire highly sensitive signals and also increase the durability of smart patches.^79^ Compared with the commercial electrodes, the advanced ionic skins could increase the signal‐noise ratio by more than 20%. Besides, to improve stability in sweat/aqueous environments, waterproof ionic skins are also developed. They are usually designed with hydrophobic polymers to expel water molecules at the skin interface and thus enhance underwater stability.^73^, ^80^ When a wireless cardiac monitoring device is integrated with the ionic skin, people could wear the ionic skin to swim, and meanwhile, the ECG and even other bio‐electrography could be remotely monitored on a smartphone (Figure 4C).^56^

**FIGURE 5:**
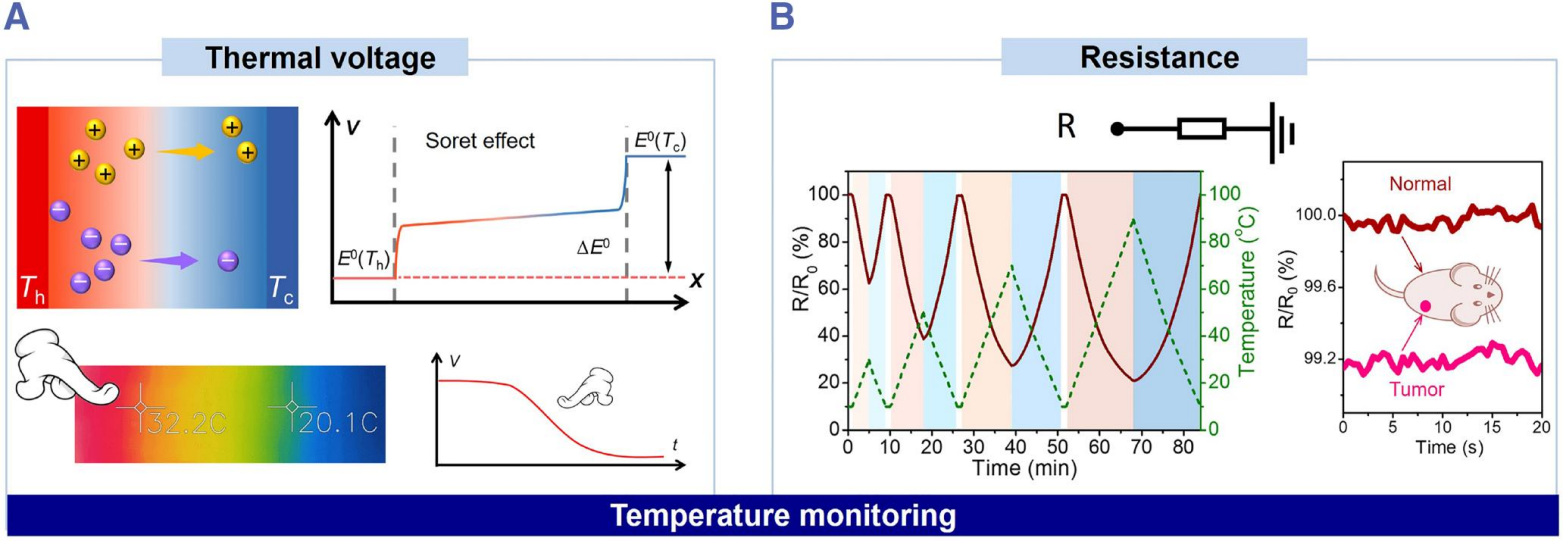
Self‐diagnostic applications of bio‐inspired ionic skins via thermal voltage and resistance. (A) Thermal diffusion of ions in temperature gradient enabling temperature sensing. (B) Temperature‐sensitive resistance enabling body temperature monitoring. Reproduced with permission.^20^ Copyright 2021, John Wiley and Sons. Reproduced under terms of the Creative Commons Attribution 4.0 International License.^33^ Copyright 2018, The Authors, published by Springer Nature.

To monitor body temperatures, there are usually two ways to acquire highly stable and sensitive ionic signals for long‐term monitoring and analysis (Figure 5). One is from thermal voltage and the other relies on resistance. The thermal voltage is generated by the thermal diffusion of ions in a temperature gradient (Figure 5A). When a high‐temperature stimulus is applied to one side of an ionic skin, the ions tend to diffuse from the hot side to the cold side because of the Soret effect.^81^, ^82^, ^83^, ^84^, ^85^, ^86^ But different ions have different diffusion abilities. For example, when more cations diffuse to and accumulate on the cold side, it results in an open‐circuit voltage from the cold side to the hot side. The temperature coefficient, which is defined as voltage per unit temperature difference, is usually a thermodynamic constant. Therefore, when the reference temperature of one side is known, the ionic skin could monitor the temperature of the other side through the thermal voltage. On the other hand, the resistance signals are related to the thermal movements of ions (Figure 5B). When the temperature increases, the migration rate of ions increases, thus decreasing the resistance of the ionic skin. Meanwhile, the resistance increases when the temperature decreases. The sensitivity is defined as the temperature coefficient of resistance (TCR), which is usually in the range of 0.01 to 0.1°C^−1^.^33^, ^77^ The human body wearing ionic skins could detect abnormal body temperature, enabling self‐diagnosis of fever and even local tumor tissues.^20^ For example, the temperature of the epidermis above tumor tissues is about 0.3° higher than that of the epidermis of normal tissues. The attached ionic skin shows a resistance change of about 0.8%, indicating an abnormal health condition.^20^

**FIGURE 6:**
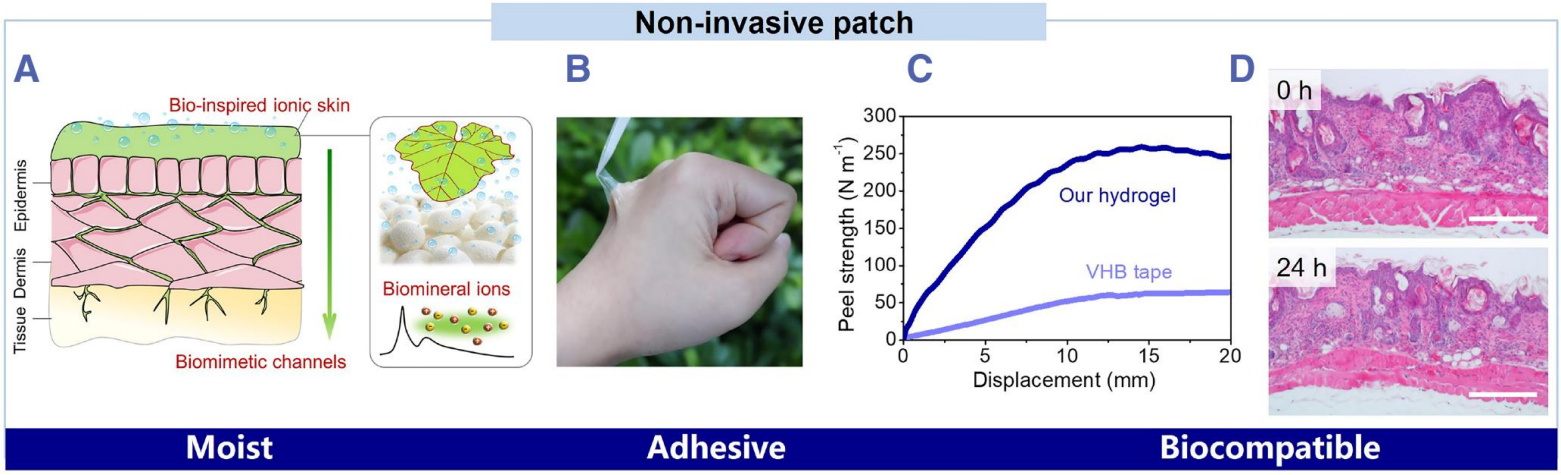
Autotherapy applications of bio‐inspired ionic skins. Reproduced with permission.^20^ Copyright 2021, John Wiley and Sons. (A) Adhesive and biocompatible ionic skins mimic the hydration nature of natural skin. (B) A photograph of the ionic skin on a human hand. (C) 90° peeling test of the ionic skin and a commercial adhesive tap (VHB). (D) Histological images of normal mouse skin before (top) and after (bottom) wearing the ionic skin for 24 h. Scale bar: 200 μm.

In addition to acquiring physiological signals for self‐diagnosis, ionic skins could serve as a local drug pump by fabricating a hydrating and physiological‐like environment on natural skin, enabling noninvasive transdermal drug delivery. To keep the ionic skin hydrating in the air, natural moisturizing factors are added.^20^ The moisturizing factors maintain a delicate balance between the evaporation and absorption of free water in an open condition, providing a hydrating and physiological‐like environment. As a result, the ionic skin could continuously monitor the human body's physiological electrical signals and enable drug delivery via an osmotic gradient in vitro. In a recent example, a biocompatible, adhesive, and hydrating ionic skin show the capacity to combine self‐diagnosis and auto‐therapy for healthcare applications.^20^ It is physically cross‐linked by a betaine analog, silk fibroin, and biomineral calcium ions (Figure 6A,B). When the ionic skin is attached to natural skin, it reaches a peel strength of about 250 N m^−1^ and maintains the skin moist (Figure 6C). After 24 h, there is no allergic reaction and the natural skin keeps elastic and hydrated (Figure 6D).

**FIGURE 7:**
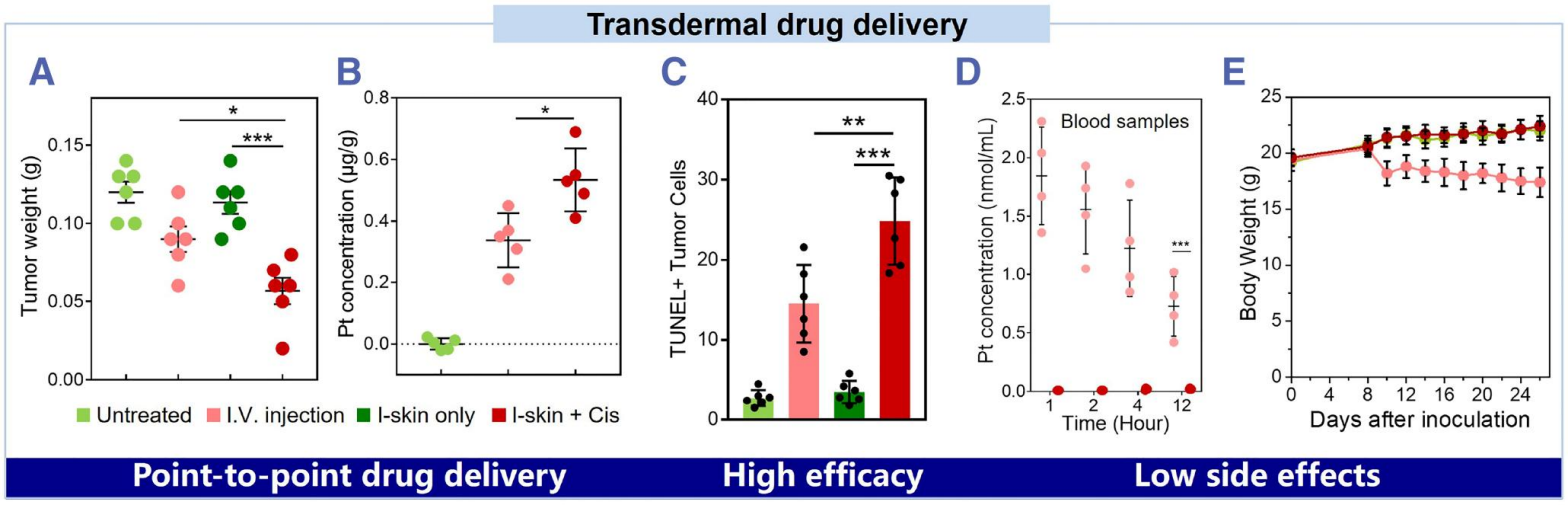
Point‐to‐point drug delivery via an osmotic gradient enables noninvasive treatment of high efficacy and low side effects. Reproduced with permission.^20^ Copyright 2021, John Wiley and Sons. (A) The weight of the subcutaneous xenografts after different treatments. (B) The cisplatin levels in tumors after different treatments. (C) The percentages of TUNEL+ cells. (D) The cisplatin levels in the blood after different treatments. (E) The body weights of mice under different treatments. The arrows indicate the cisplatin treatment via I.V. injection.

Therefore, drug‐loaded biocompatible ionic skins can directly promote surface wound healing.^87^, ^88^, ^89^ Meanwhile, for the disease inside of the body, the ionic skin could also automatically release drugs and point‐to‐point delivery to the inner tissue through a concentration difference between the ionic skin and the human skin. For example, the ionic skin is loaded with water‐soluble cisplatin to treat the tumor tissues (Figure 7). Compared with the direct injection of cisplatin, the theranostic ionic skin enables a higher concentration of local enrichment of the drugs in the tumor tissues while decreasing the drug concentration in the blood (Figure 7A–D). The mice treated with drug‐loaded ionic skins show increasing body weight, indicating good health conditions (Figure 7E). To further improve the on‐demand drug delivery efficiency, drug carriers and exogenous stimuli could be combined.^90^, ^91^ Overall, ionic skins not only open a new opportunity for facile diagnosis and therapy but also improve treatment efficacy and reduce the side effects of major diseases. With the development of integration techniques and data processing ability in ionic skins, remote smart healthcare is foreseeable.

## 6 CHALLENGES AND OPPORTUNITIES

**FIGURE 8:**
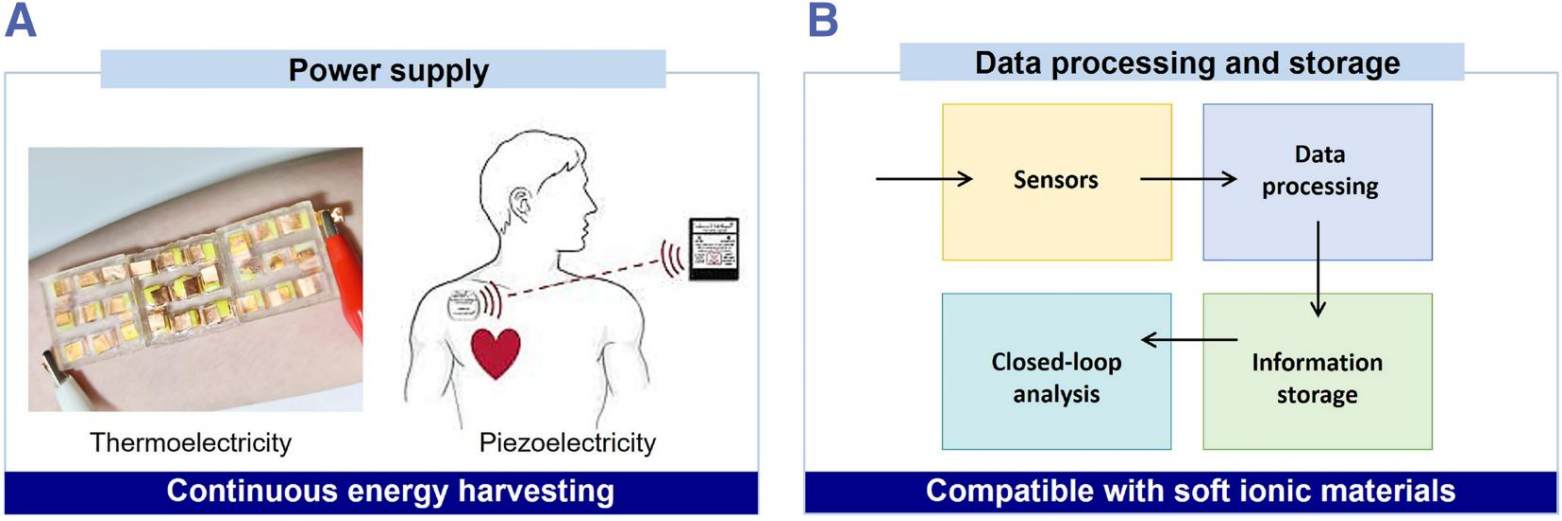
Challenges and opportunities of bio‐inspired ionic skins. (A) Reliable and sustainable power supplies that could harvest waste energy from the human body. (B) Integrating compatible data processing and storage units for soft ionic systems.

Despite the achievements within the past few years, ionic skin is still in its infancy state. There are two significant challenges: (1) lacking a high‐efficient and continuous power supply for ionic skins and (2) lacking integration of data processing and storage components (Figure 8).

Lithium batteries have been widely used in personal electronic devices, but they rarely meet the requirements of ionic skin, that is, softness and stretchability. Aqueous metal‐ion batteries can be soft and stretchable, but they have relatively small capacity and unstable long‐term performance.^92^ Other energy harvesting and conversion techniques, such as solar cells, require additional energy sources for electricity harvesting. A sustainable energy conversion mode is harvesting the body's motion and thermal energy into electricity. In this regard, triboelectric, piezoelectric, thermoelectric, and hybrid energy conversion technologies are promising candidates.^32^, ^93^, ^94^, ^95^, ^96^ However, the generation of triboelectricity depends on a high frequency of motion, which is difficult to realize when people relax and sleep. Meanwhile, the energy conversion efficiency of existing piezoelectric and thermoelectric materials is relatively low and thus requires a series integration of multiple high‐conductive modules to boost the output power.

On the other hand, the data processing and storage components for the ionic skins are mainly based on conventional printed circuit boards and rigid semiconductor electronics, causing extra burdens for the ionic skin systems. One possible opportunity is to develop circuit boards based on liquid metals and stretchable semiconductive polymers.^97^, ^98^, ^99^, ^100^, ^101^ However, the emergence of electric‐double‐layer interfaces in ionic skin systems may increase power consumption and decrease efficiency. In contrast, the biological system combines the intelligence of stimuli perception, signal transmission, signal processing, and information storage. All of the functions are performed by ionic signals. This is because the biological system has unique logic manners to control and tune the ionic signals. It is a crucial challenge for an artificial system to mimic the biological manner to process the ionic signals, thus enabling to merge the gap among the sensing, processing, and storage components. Recently, a trimetric design in artificial hydrogels is proposed to mimic the cell membrane structure and the resting potential to meditate their ionic signals.^102^ The trimetric hydrogels demonstrate the potential to combine the intelligence of sensing external stimuli, encoding logical responses, synapse‐like plasticity, and even multistore image memory. In the future, it is important to improve the efficiency of signal processing and the maturity of logical response.

## 7 CONCLUSION

This review first discusses the molecular design for bio‐inspired ionic skins to improve mechanical adaptability. The development of various hydrogels and the continuous optimization of their molecular structures enable the rapid development of ionic skins. The state‐of‐the‐art ionic skins achieve perfect mechanical adaptability on the human skin and could acquire physiological signals of the human body through diverse device configurations. Compared with implantable devices, ionic skins provide another safe way for future smart medicine. However, the development of ionic skins is still in the early stage. Through the integration of data processing and storage components and power supplies, more smart functions and applications of ionic skins can be developed for future self‐diagnosis and on‐demand drug delivery. We believe this will reshape the future of smart medicine.

## AUTHOR CONTRIBUTIONS

Zhouyue Lei and Guogao Zhang conceived the idea and designed the review. All the authors wrote, discussed and commented the review.

## CONFLICT OF INTEREST STATEMENT

The authors declare no conflict of interest.
